# Cholinergic signaling mediates the effects of xenin-25 on secretion of pancreatic polypeptide but not insulin or glucagon in humans with impaired glucose tolerance

**DOI:** 10.1371/journal.pone.0192441

**Published:** 2018-02-21

**Authors:** Songyan Wang, Lauren Z. Oestricker, Michael J. Wallendorf, Karin Sterl, Judit Dunai, C. Rachel Kilpatrick, Bruce W. Patterson, Dominic N. Reeds, Burton M. Wice

**Affiliations:** 1 Department of Internal Medicine, Division of Endocrinology, Metabolism and Lipid Research Washington University School of Medicine, Saint Louis, MO United States of America; 2 Division of Biostatistics, Washington University School of Medicine, Saint Louis, MO United States of America; 3 Department of Internal Medicine, Division of Nutritional Science, Metabolism and Lipid Research Washington University School of Medicine, Saint Louis, MO United States of America; University of Miami Miller School of Medicine Palm Beach Regional Campus, UNITED STATES

## Abstract

We previously demonstrated that infusion of an intestinal peptide called xenin-25 (Xen) amplifies the effects of glucose-dependent insulinotropic polypeptide (GIP) on insulin secretion rates (ISRs) and plasma glucagon levels in humans. However, these effects of Xen, but not GIP, were blunted in humans with type 2 diabetes. Thus, Xen rather than GIP signaling to islets fails early during development of type 2 diabetes. The current crossover study determines if cholinergic signaling relays the effects of Xen on insulin and glucagon release in humans as in mice. Fasted subjects with impaired glucose tolerance were studied. On eight separate occasions, each person underwent a single graded glucose infusion- two each with infusion of albumin, Xen, GIP, and GIP plus Xen. Each infusate was administered ± atropine. Heart rate and plasma glucose, insulin, C-peptide, glucagon, and pancreatic polypeptide (PP) levels were measured. ISRs were calculated from C-peptide levels. All peptides profoundly increased PP responses. From 0 to 40 min, peptide(s) infusions had little effect on plasma glucose concentrations. However, GIP, but not Xen, rapidly and transiently increased ISRs and glucagon levels. Both responses were further amplified when Xen was co-administered with GIP. From 40 to 240 min, glucose levels and ISRs continually increased while glucagon concentrations declined, regardless of infusate. Atropine increased resting heart rate and blocked all PP responses but did not affect ISRs or plasma glucagon levels during any of the peptide infusions. Thus, cholinergic signaling mediates the effects of Xen on insulin and glucagon release in mice but not humans.

## Introduction

Xenin-25 (Xen) is a 25-amino acid neurotensin-related peptide produced by a subset of enteroendocrine cells [1,2]. In animals, Xen administration delays gastric emptying [3], reduces food intake [4–6], increases intestinal motility [7], augments gall bladder contractions [8], enhances exocrine pancreas secretion [9], and excites a small subset of enteric neurons [10]. We [11] and others [12] have shown that Xen amplifies the effects of glucose-dependent insulinotropic polypeptide (GIP) on insulin secretion in mice. Surprisingly, this in vivo response to GIP plus Xen was not recapitulated in studies using isolated islets, insulin-producing cell lines, or the in situ perfused [11]. However, the ability of Xen to amplify GIP-mediated insulin secretion in vivo was inhibited by atropine. Additionally, in vitro experiments showed that the in vivo effects of Xen could be mimicked by carbachol. These results indicate that in mice, Xen initiates a cholinergic relay to islets which in turn, amplifies the effects of GIP on insulin release [11].

Many of the effects of Xen are mediated by activation of neurotensin receptor-1 on neurons [3,5,6,8,10,13]. Immunohistochemical studies showed that in the human pancreas, neurotensin receptor-1 is present on nerves, but not islet endocrine cells [14]. In human studies, we showed that Xen delays gastric emptying [15], increases pancreatic polypeptide release [14,16], inhibits glucagon-like peptide-1 release [15], augments intestinal motility [15,17], and amplifies the effects of GIP on insulin, glucagon, and pancreatic polypeptide release [14,17]. Contrary to dogma, these studies also demonstrated that exogenously administered GIP remained fully active in humans with mild type 2 diabetes mellitus [17]. Conversely, the effects of Xen on GIP-mediated insulin and glucagon release were greatest in humans with impaired glucose tolerance but blunted in those with mild type 2 diabetes mellitus [17]. Similarly, we previously showed that the effect of Xen on GIP-mediated insulin secretion was greater in hyperglycemic compared to normoglycemic mice [11]. These collective results suggest that increased cholinergic signaling is a compensatory neural mechanism to increase insulin secretion in pre-diabetes and type 2 diabetes develops if this adaptation fails. Thus, it is critical to determine if the effects of Xen on insulin and glucagon release are mediated by cholinergic signaling in humans as in mice. The purpose of the present study is to determine if atropine inhibits the ability of Xen to amplify the effects of GIP on insulin, glucagon, and pancreatic polypeptide release in humans. As expected, atropine completely blocked the pancreatic polypeptide response to GIP alone, Xen alone, and the combination of GIP plus Xen. Surprisingly and in stark contrast to mice, the effects of Xen on GIP-mediated insulin and glucagon release were not inhibited by atropine and thus, are not mediated by cholinergic signaling.

## Materials and methods

### Human subjects

All protocols were approved by Washington University’s Human Research Protection Office (November 12, 2012) and the FDA (IND#103,374) and are registered with ClinicalTrials.gov (NCT01951729). The original study protocol as approved by the IRB is presented in S1 Appendix. An administrative error resulted in a delay in registering this clinical trial (all ongoing and related studies are currently registered). Subjects were recruited through Washington University’s Research Participant Registry, our own database, and by visibility on the Clinicaltrials.gov website. Studies were performed by the nursing and medical staff in the Clinical Research Unit of the Institute of Clinical and Translational Sciences of Washington University after obtaining written informed consent. As part of the consent, participants agreed that if we write a report or article about this study or share the study data set with others, we will do so in such a way that they cannot be directly identified. Subject recruitment was initiated on March 13, 2013 and follow-up for the final participant was completed on May 13, 2015. Subjects were remunerated to encourage completion of the study. An initial screening visit was conducted after a 10-hour overnight fast. Subjects completed a health history questionnaire, underwent an EKG, and had blood drawn for screening labs including hematocrit, hemoglobin, HbA1C, lipid panel, electrolytes, amylase, and thyroid, liver and kidney function. Subjects were excluded if they had a history of chronic pancreatitis and/or risk factors for chronic pancreatitis, had a history of gastrointestinal disorders, were taking any medication known to affect glucose homeostasis, or had significant systemic illness including heart, kidney, liver, inflammatory or malignant disease. The use of atropine is contraindicated in those with narrow-angle glaucoma, obstructive uropathy including benign prostatic hypertrophy, pyloric stenosis, myasthenia gravis, asthma, hyperthyroidism, angina and cardiac arrhythmias including heart block. Subjects with any of these conditions were also excluded. Subjects who were otherwise eligible received a standard 75-gram oral glucose tolerance test on a subsequent visit to determine final eligibility. Impaired glucose tolerance was defined by the 2-hour plasma glucose level (141 to 199 mg/dl) during the oral glucose tolerance test using diagnostic criteria of the American Diabetes Association [18]. Male and female subjects with impaired glucose tolerance, 18 to 65 years of age, and of all races and ethnicities were eligible. Women of childbearing potential were required to use effective birth control.

### Study design

**Fig 1:**
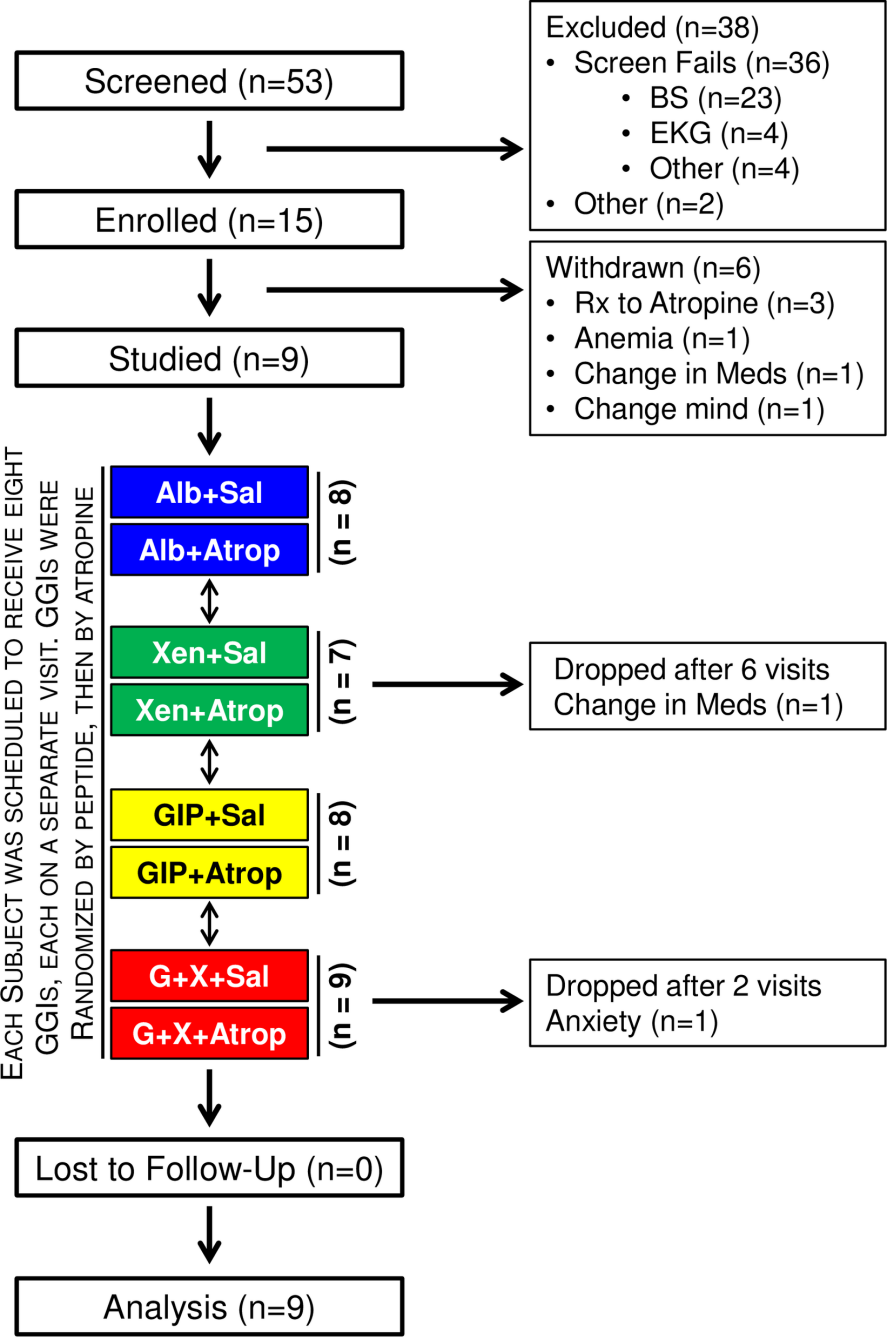
Flow diagram for atropine study. The flow diagram is for a crossover study in humans with impaired glucose tolerance and was designed so that each subject would receive all 8 graded glucose infusions. The same peptide(s) was administered during 2 successive visits- once with saline and once with atropine. First, the order of the peptide infusions was randomized. Second, the order of saline or atropine administration was randomized for each peptide. For example, if the subject was randomized to first receive Xen alone, this person would receive Xen in study visits 1 and 2. The atropine and saline infusions would then be randomized to visit 1 and 2. Eight subjects received all 8 graded glucose infusions. One subject received 6 infusions but did not receive either infusion with Xen alone. One subject received only the 2 infusions with GIP plus Xen.

**Fig 2:**
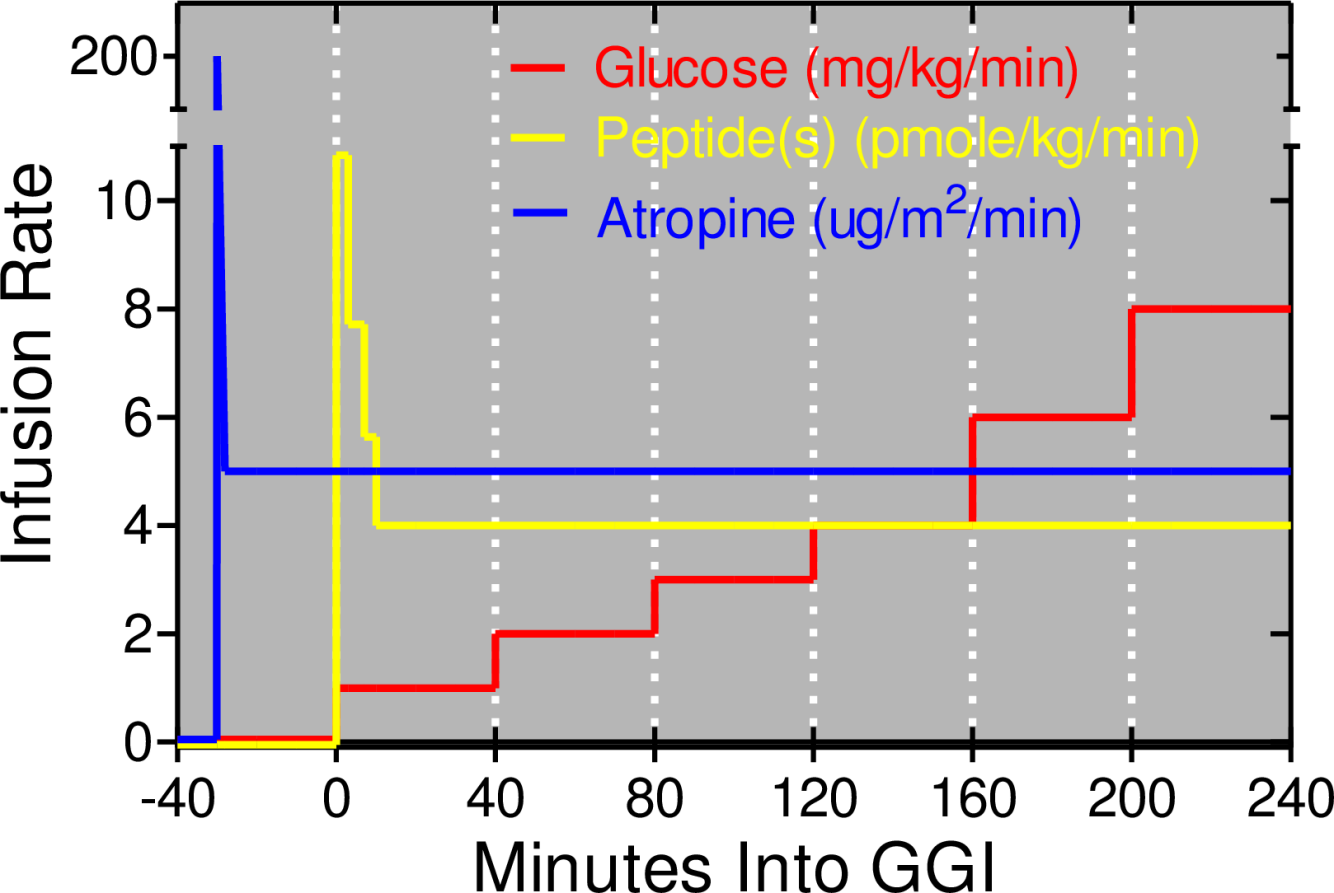
The graded glucose infusion protocol. The glucose infusion rate was increased in a step wise fashion every 40 minutes starting at time zero as shown in red. The primed-constant infusion of each peptide(s) was started at time zero and is shown in yellow. Note that GIP and Xen were each infused at the same rate when administered together. The primed-constant infusion of atropine (or saline) was initiated 30 minutes before the start of the graded glucose infusion as shown in blue. All infusions were terminated 240 minutes after the glucose infusion was initiated.

This is a crossover study in which each participant was to undergo 8 separate 240-minute graded glucose infusions, each separated by at least 2 weeks (Fig 1). Studies were performed after a 10-hour overnight fast. Subjects were blinded to treatment. One intravenous catheter was placed into a hand vein. This hand was kept in a thermostatically controlled box (50–55°C) to facilitate venous sampling and to provide arterialized venous blood [19]. A second intravenous line was inserted for administration of glucose and study drugs. For each graded glucose infusion (Fig 2), the intravenous glucose infusion was initiated at time zero and maintained at a rate of 1 mg x kg^-1^ x min^-1^ for 40 min, followed by 2, 3, 4, 6, and 8 mg x kg^-1^ x min^-1^ (40 min for each step) as in our earlier study [17]. Albumin alone (i.e. no peptide), Xen alone, GIP alone, and GIP+Xen were administered by primed-constant infusions starting at time zero as previously described [17]. Briefly, a continuous infusion of peptide(s) was maintained at a rate of 4 pmoles x kg^-1^ x min^-1^ throughout the experiment. However, a priming dose was administered during the first 10 minutes by increasing the infusion rate 2.71-fold for the first 3 minutes, 1.93-fold for the next 4 minutes, and 1.41-fold for the final 3 minutes. Albumin alone was infused at the same rate when peptides were not administered. Each peptide(s) was administered both with and without an infusion of atropine sulfate. Dosing for atropine sulfate was based on a survey of the literature [20–31] and studies registered on the Clinicaltrials.gov website (NCT00468091; NCT00689208; and NCT00992901). Atropine sulfate was administrated by primed-constant intravenous infusion starting at minus 30 minutes (priming dose of 0.4 mg/m^2^ over 2 minutes followed by continuous dose of 0.3 mg/m^2^/hour). Saline alone was infused when atropine was not administered. Infusion rates for glucose, peptides, and atropine/saline are shown in Fig 2. The same peptide(s) was administered in 2 successive visits. Atropine was administered with the peptide on one visit and saline (instead of atropine) was administered with the same peptide on the subsequent visit. The order of the paired visits was first randomized with respect to the peptide(s) after which the order of the atropine or saline infusion was randomized. This was done to ensure that in case of drop outs, matched infusions (each peptide ± atropine) would be obtained for each subject. Hemoglobin levels were measured immediately before each study visit and anyone with a Hb <11.2 g/dL had that study delayed.

### Peptides

GIP and Xen were custom synthesized under GMP conditions (Bachem, Torrance, CA), validated for use in humans, and compounded in normal saline containing 1% Flexbumin (Baxter Healthcare Corp., Westlake Village, CA) as previously described [17].

### Blood sampling

Blood was drawn for measurements of plasma glucose and preparation of heparinized plasma at the following time points (in minutes): -40, -30, -20, -10, 0, 5, 10, 15, 20, 30, 40, 50, 60, 70, 80, 90, 100, 110, 120, 130, 140, 150, 160, 170, 180, 190, 200, 210, 220, 230, 240. Blood was collected for preparation of EDTA/Trasylol plasma at -40, -30, -20, -10, 0, 5, 10, 15, 20, 30, 40, 80, 120, 160, 200, and 240 minutes. A DPP4 inhibitor (Millipore, St. Charles, MO) was also included in the EDTA/Trasylol tubes.

### Assays

Glucose, insulin, C-peptide, Xen, total GIP, complete metabolic profiles and hemoglobin A1c were measured as previously described [32]. Glucagon was measured as previously described [32] using an ELISA specific for mature glucagon that does not cross-react with other proglucagon-derived peptides. Pancreatic polypeptide was measured as previously described and involves an extraction step to remove compounds that interfere with the assay [14].

### Calculations and data analysis

ISRs were derived by stochastic deconvolution of the peripheral C-peptide concentrations as in earlier studies [15–17,32] using population-based estimates of C-peptide clearance kinetics [33–35]. Baseline values for glucose, ISR, and pancreatic polypeptide are mean values for the -50, -40, -30 time points. Baseline for glucagon are mean values of -30, -20, and -10 time points because baseline values continually dropped from -50 to -30 minutes and the decline was not affected by atropine. Baseline levels for each individual were used for their respective calculations. AUCs were calculated for each individual using the trapezoid method and incremental AUCs were determined by subtracting that individual’s baseline AUC from the AUC. Data for AUCs and iAUCs were analyzed using mixed effects models with subject as a random effect and treatment as a fixed effect using SAS v9.4. Baseline values were used as a covariate for the analysis of the AUCs. Data for the time frames of 0 to 40 minutes and 40 to 240 minutes were analyzed separately because the former represents a rapid, large, and transient response and the latter is a progressively changing effect. Thus, the early and late responses may be regulated by distinct mechanisms. Outcome measures through time were analyzed using the mixed random effects repeated measures model with covariance structure estimated by a spatial model (SAS 9.4). Subject and subject by drug interaction were random effects. Individual data points used for calculating means, (incremental) areas under the curve, and variance measures are provided in the S2 Appendix.

## Results

### Subject characteristics

We previously demonstrated [17] that the ability of Xen to amplify the effects of GIP on insulin and glucagon release is greatest in humans with impaired glucose tolerance and therefore, only subjects with impaired glucose tolerance were studied. Fifty three subjects were screened and 15 were enrolled and studied on at least 1 occasion (Fig 1). Most screen failures were due to subjects not meeting the criteria for impaired glucose tolerance (see below) or having a contraindication for receiving atropine. Six patients were withdrawn from the study and data from these participants were not included in the analyses. Data from the 9 remaining subjects were analyzed. Clinical characteristics and demographics for these 9 subjects are shown in Table 1. Seven of the participants completed all 8 visits. One subject was dropped before both Xen infusions due to change in medication and one was dropped after both GIP plus Xen visits due to anxiety. Consistent with the screening protocol, all subjects had impaired glucose tolerance based on the 2-hour glucose value from a standard 75-gram oral glucose tolerance test.

**Table 1:** Subject demographics and clinical characteristics.

*Parameter*	*n = 9*
2-Hour Glucose (mg/dl)	170± 20
HbA1c (%)	6.0 ± 0.4
Fasting Glucose (mg/dl)	99.3 ± 7.8
Fasting Insulin (μU/ml)	11.4 ± 4.9
HOMA-IR	2.8 ± 1.3
BMI, kg/m^2^	30.4 ± 8.2
Age (years)	46 ± 10
Gender (F/M)	6/2

Values except gender are group mean ± SD.

### Peptide levels during infusion of GIP and/or Xen

**Fig 3:**
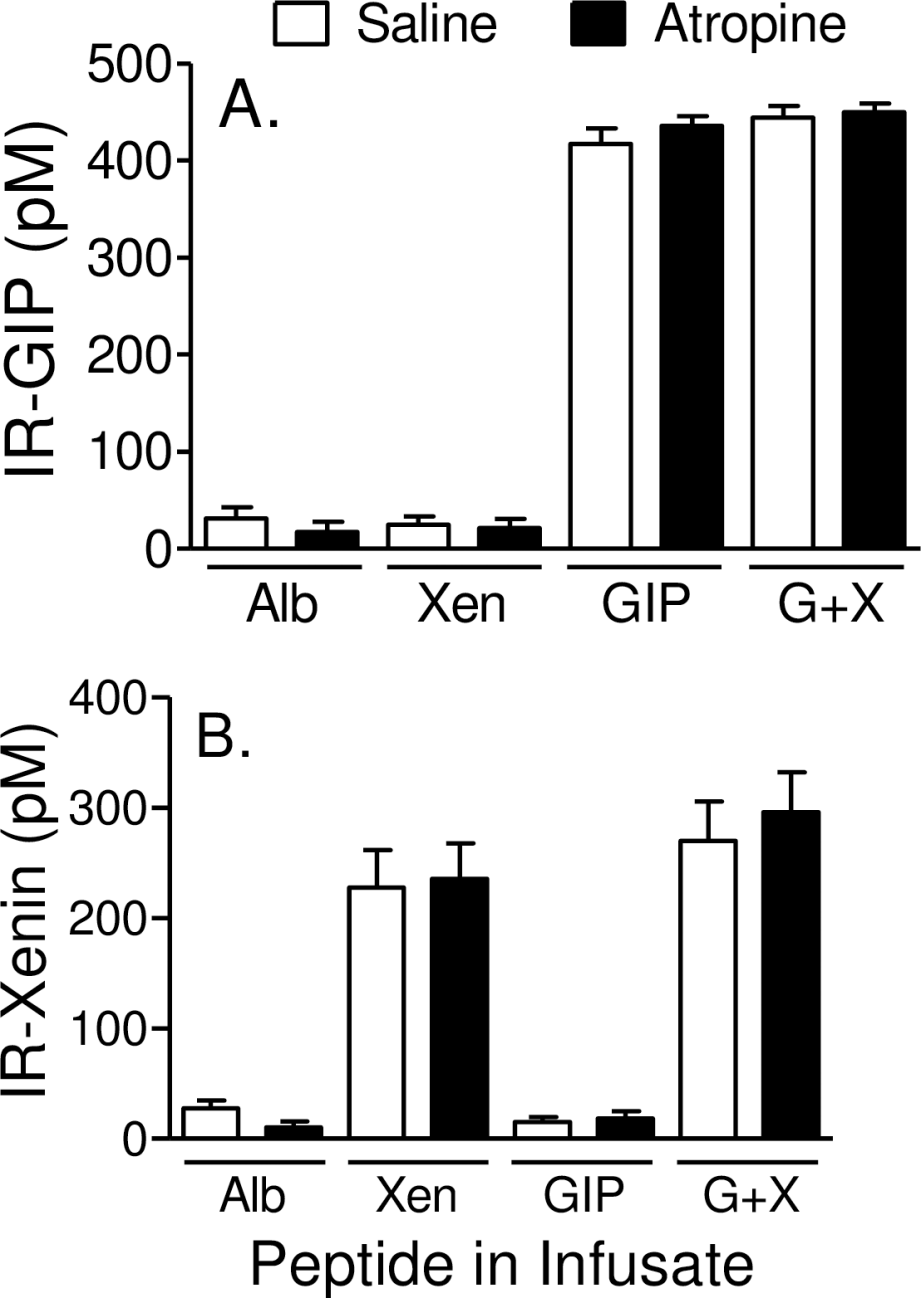
Peptide levels during graded glucose infusions. Subjects were administered 8 different graded glucose infusions, each on a separate day. Each visit was separated by at least 2 weeks. Glucose and peptides were infused from 0 min to 240 min and atropine (or saline control) was infused from -30 to 240 min as shown in Fig 2. Steady state levels of immunoreactive-GIP (IR-GIP; panel A) and immunoreactive-Xen (IR-Xen; panel B) were measured during infusion with albumin alone (Alb), Xen alone, GIP alone, and the combination of GIP plus Xen (G+X). Each peptide was measured during infusion of atropine or the saline control. Because of limiting sample volumes, GIP and Xen were measured only in the 80 or 240 minute samples, respectively. Values represent group means ± SEM.

Steady-state levels of GIP and/or Xen are attained approximately 10 minutes after starting the primed-constant peptide infusions and are maintained until the end of the graded glucose infusion [17]. As in our earlier study, infusion of GIP increased steady state levels of plasma GIP from ~25 pM to ~400 pM whereas infusion of xenin-25 increased plasma xenin levels from the lower limit of detection to ~200 pM (Fig 3). Infusion of one peptide did not affect plasma levels of the other. Further, infusion of atropine did not affect plasma levels of either peptide. Thus, pharmacologic levels of GIP and/or Xen were attained throughout the graded glucose infusions.

### Atropine increases resting heart rate

**Fig 4:**
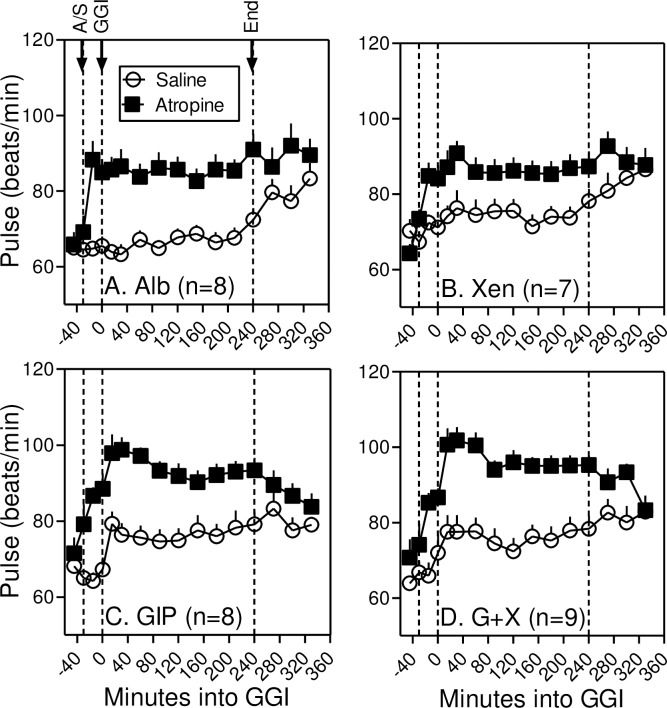
Atropine increases resting heart rate. Resting heart rate was measured at the indicated times before, during and after the graded glucose infusion (GGI) with Alb alone (Panel *A*), Xen alone (Panel B), GIP alone (Panel C), and GIP plus Xen (G+X; Panel D). Atropine or saline infusion was started 30 minutes before the graded glucose infusion. Note that atropine or GIP increased resting heart and values were normalized approximately 90 minutes after the graded glucose infusions were terminated. After 240-minutes, heart rates increase in the subjects administered saline instead of atropine because they are no longer confined to the bed. Values represent group means ± SEM.

In the absence of atropine, infusion of Alb alone or Xen alone had little effect on resting heart rate (Fig 4). As in our earlier study [17], infusion of GIP alone increased resting heart rate by ~9 beats per minute and this response was not amplified when Xen was administered along with GIP. Infusion of atropine increased resting heart rate before peptides were administered (i.e. from -30 to 0 minutes) as well as after the peptide infusions were initiated. Heart rate was normalized ~90 minutes after the atropine infusion was terminated. Mean arterial pressures were unaffected by atropine (Not Shown) although infusion of GIP, alone or with Xen, decreased mean arterial pressure by ~10 mm Hg as in our earlier study [17]. Based on symptom surveys self-reported by subjects before, during, and after the infusions, xerostomia was experienced by all subjects during 1 or more of the 4 atropine infusions (mean = 2.6; SD = 1.1). Blurred vision was self-reported by 3 of the 9 subjects during 2 (n = 2) or 3 (n = 1) of the atropine infusions. These results indicate that atropine dosing was effective in our patient population.

### Peptides increase cholinergic signaling in humans

**Fig 5:**
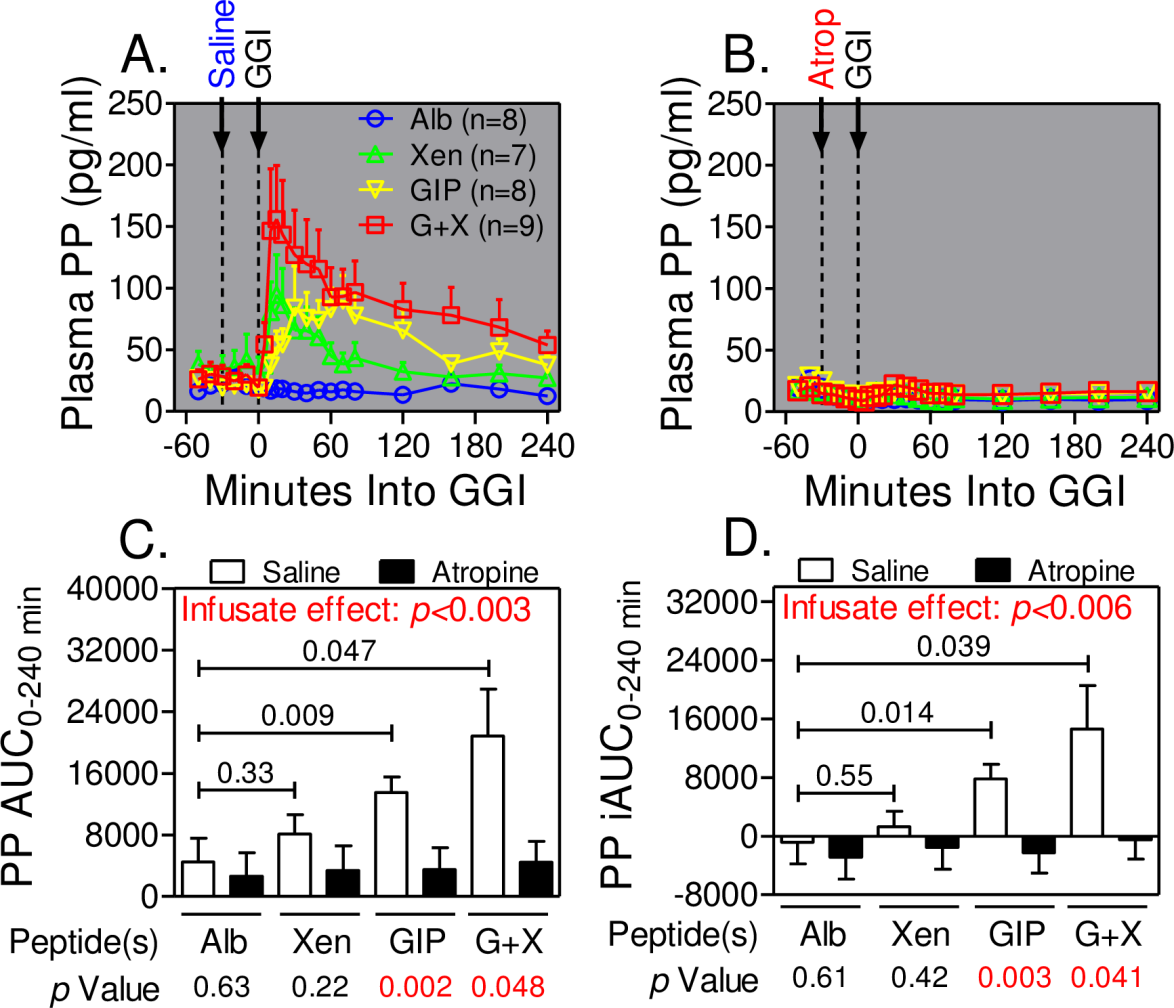
Atropine inhibits pancreatic polypeptide release. Panels A and B. Pancreatic polypeptide (PP) levels were measured at the indicated times before and during graded glucose infusions (GGIs) in the presence of the indicated peptide(s) and with infusion of saline (Panel A) or atropine (Panel B). Total (Panel C) and incremental (Panel D) AUCs from 0–240 minutes were calculated from data in panels A and B. Incremental and total AUCs were determined for each individual and values represent group means ± SEM. Significance was determined using the mixed effects model. *p* values for each peptide versus albumin alone are indicated within charts in Panels C and D. *p* values for the effects of atropine for each peptide infusate (or albumin control) are indicated below Panels C and D. The *p* values for infusate effects were calculated for all 8 treatments. Note that atropine infusion completely blocks all pancreatic polypeptide responses.

As reported earlier [14], infusion of peptide(s) alone, but not albumin alone, rapidly increased pancreatic polypeptide concentrations after which the levels slowly declined but remained elevated for the duration of the graded glucose infusion (Fig 5A). The pancreatic polypeptide response was greatest during infusion of GIP+Xen compared to GIP alone and Xen alone. The pancreatic polypeptide response to GIP was delayed compared to that with Xen alone. All peptide-dependent increases in pancreatic polypeptide levels were abolished when atropine was administered during the graded glucose infusion (Fig 5B). Like the temporal profiles, pancreatic polypeptide AUCs (Fig 5C) and iAUCs (Fig 5D) were also increased in the order of GIP+Xen > GIP alone >Xen alone > Alb alone and the increases were completely blocked by atropine (*p*<0.003 and <0.006 for respective infusate effects). Thus, peptide(s) infusions increase cholinergic signaling in humans.

### Cholinergic signaling does not mediate the effects of peptides on insulin secretion

**Fig 6:**
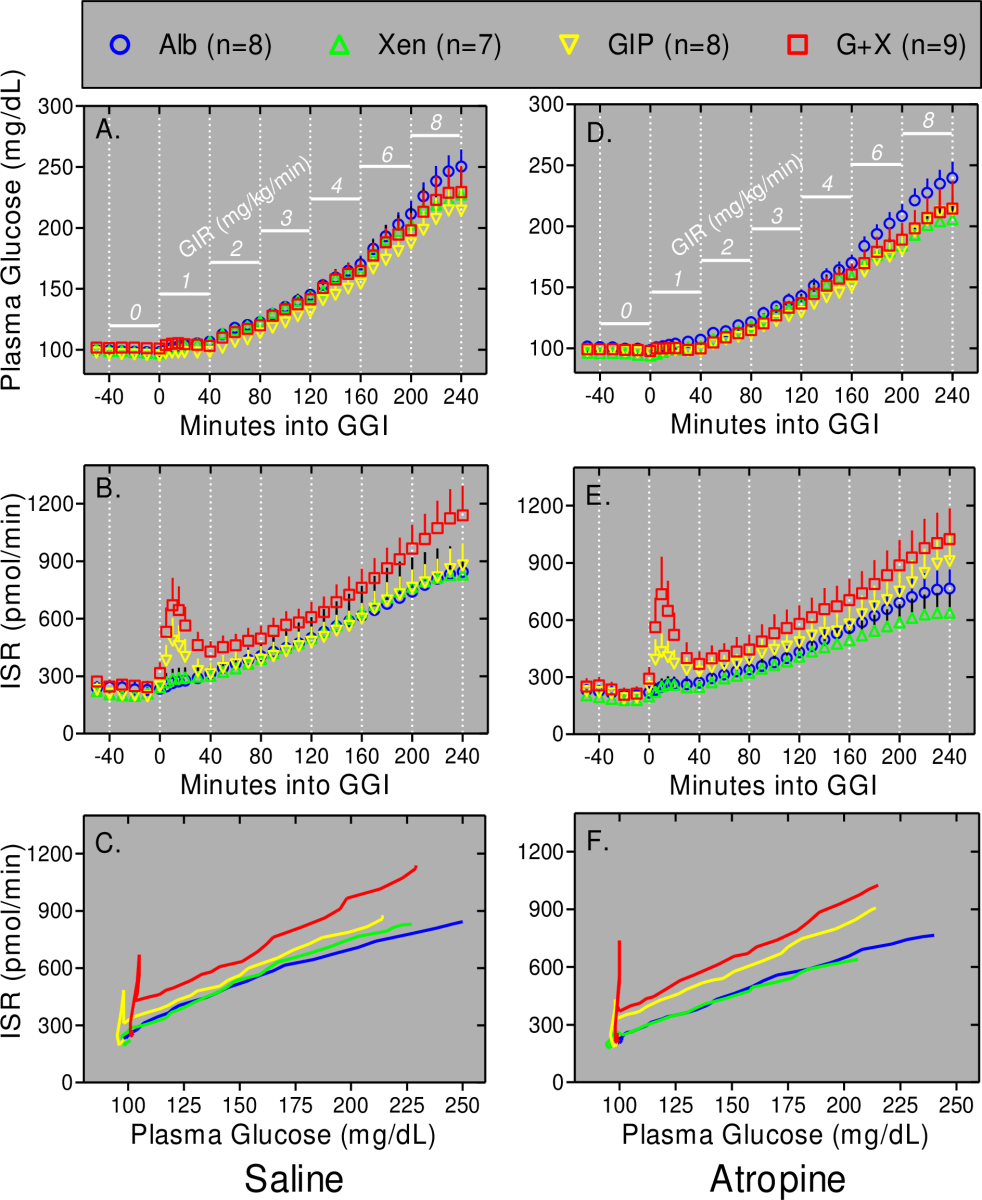
Xen amplifies the effects of GIP on ISRs. Graded glucose infusions (GGIs) with the saline control (Panels A-C) or atropine infusion (Panels D-F) were conducted. Plasma glucose (Panels A and D) and ISRs (Panels B and E) are shown for the indicated times before and during GGIs. The glucose infusion rate (GIR) at each 40 minute step is shown in white. Note that plasma glucose levels increase progressively even though glucose was administered in a step-wise fashion. Data from panels A and B are re-graphed in panel C whereas data from panels D and E are re-graphed in panel F. Symbols and error bars are eliminated in panels C and F for clarity. The rapid and transient increases in ISR in response to GIP and GIP plus Xen from 0–40 minutes (Panels B and E) are reflected by the initial spikes in ISRs (Panels C and F) that occur in the absence of a significant increase in plasma glucose levels. Values represent group means ± SEM.

**Fig 7:**
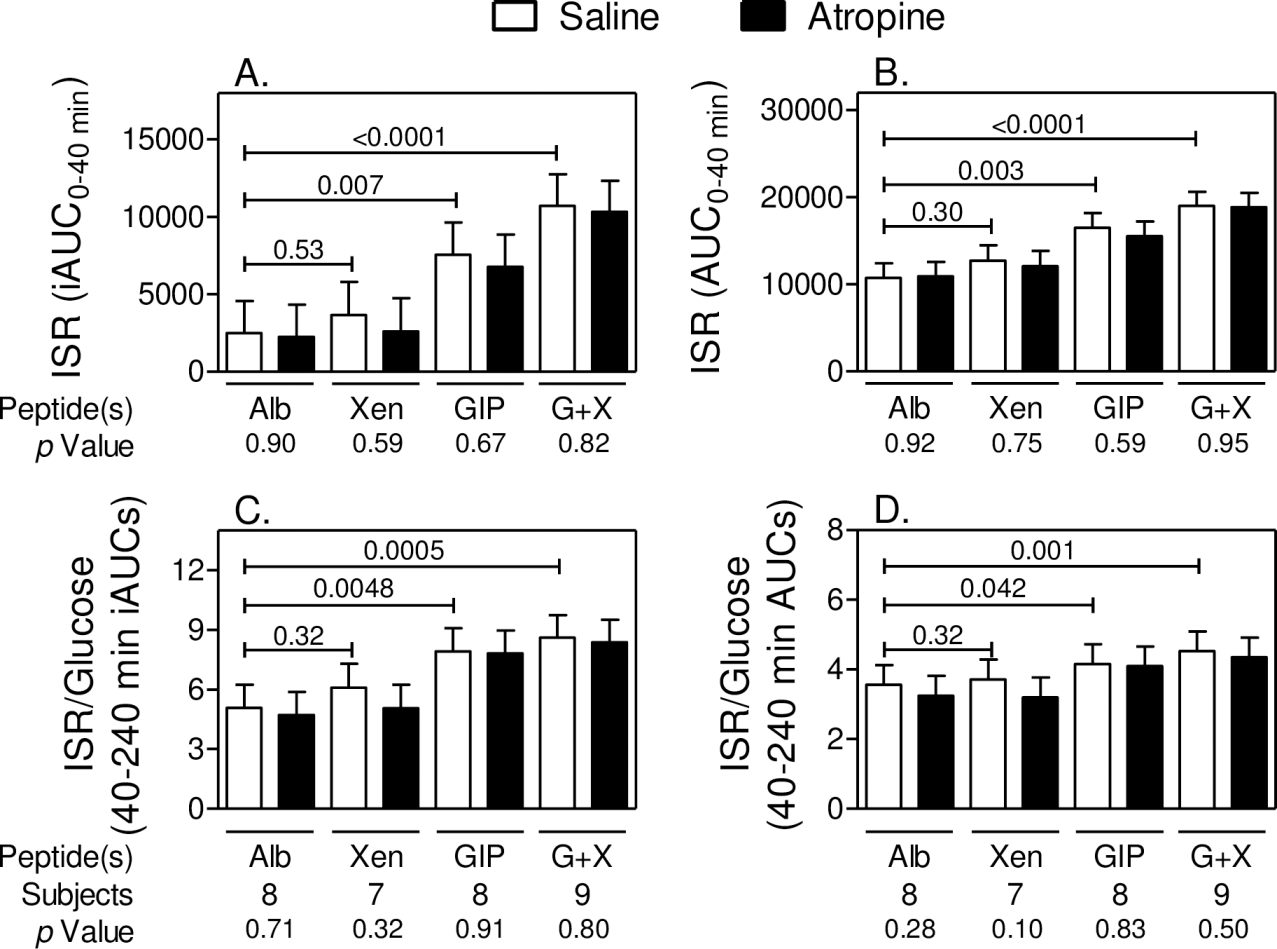
Atropine does not inhibit effects of peptides on ISRs. Incremental (Panels A and C) and total (Panels B and D) AUCs were calculated using data shown in Fig 6. Baseline values (average from -50 to -30 min) were subtracted to calculate incremental AUCs. Outcomes were determined for each individual and values represent group means ± SEM. Significance was determined using the mixed effects model. Panels A and B: Data are for 0–40 min (Panels *A* and *B*). Panels C and D: Data are for 40–240 min. Values were calculated from the 40 to 240 ISR (i)AUCs for each individual divided by the 40 to 240 glucose (i)AUCs for the same individual. Values represent group means ± SEM.

As in our earlier study [17], plasma glucose levels remained nearly euglycemic for the first 40 minutes of the graded glucose infusion in the presence or absence of peptide(s) (Fig 6A). In contrast, there was a rapid, large, and transient increase in ISRs over this same period during infusion of GIP alone, but not Xen alone, and this transient response was even greater when Xen was administered along with the GIP (Fig 6B). These early insulin secretory responses occur in the absence of a significant increase in plasma glucose levels (Fig 6C). The rapid and transient increases in ISRs were quantified by determining AUCs for ISRs for the time frame of 0–40 min. In agreement with the temporal data in Fig 6, both the incremental (Fig 7A) and total (Fig 7B) AUCs were respectively increased 3-fold (*p* = 0.007) and 1.5-fold (*p* = 0.003) by infusion of GIP alone, but not Xen alone. Administration of the combination of GIP plus Xen increased the early ISR response for both incremental (4.3-fold) and total (1.8-fold) AUCs; (*p*<0.0001 for both; Fig 7A and 7B). Over this first 40 minutes, the effects of peptides on the profiles for plasma glucose levels and ISRs were similar with (Fig 6D–6F) and without (Fig 6A–6C) infusion of atropine. Similarly, the ISR iAUCs and AUCs for each peptide over the first 40 minutes of the graded glucose infusions were unaffected by atropine (Fig 7A and 7B). These results indicate the rapid and transient insulin secretory response to peptides is not mediated by cholinergic signaling.

After the first 40 minutes of the graded glucose infusions, both plasma glucose (Fig 6A and 6D) and ISRs (Fig 6B and 6E) progressively increased such that ISR as a function of plasma glucose increased linearly in the order of GIP+Xen > GIP alone > xenin alone or albumin alone (Fig 6C and 6F). Because peptides affected the 40 minute values for ISR versus plasma glucose (Fig 6C and 6F), the later ISR responses were quantified by determining incremental and total AUCs for ISR and plasma glucose levels (from 40–240 min for both outcomes) and then calculating the ratio of ISR to glucose. Results revealed that infusion with GIP alone, but not Xen alone, amplified the ISR/glucose incremental (Fig 7C) and total (Fig 7D) AUCs 1.55-fold (*p* = 0.0048) and 1.17-fold (*p* = 0.042), respectively and amplification was further increased to 1.70-fold (*p* = 0.0005) and 1.27-fold (*p*<0.001), respectively by infusing the combination of GIP plus Xen. The 40 to 240 minute ISR/glucose responses to peptides were not inhibited by atropine indicating that they are not mediated by cholinergic signaling.

### Cholinergic signaling does not mediate the effects of peptides on glucagon levels

**Fig 8:**
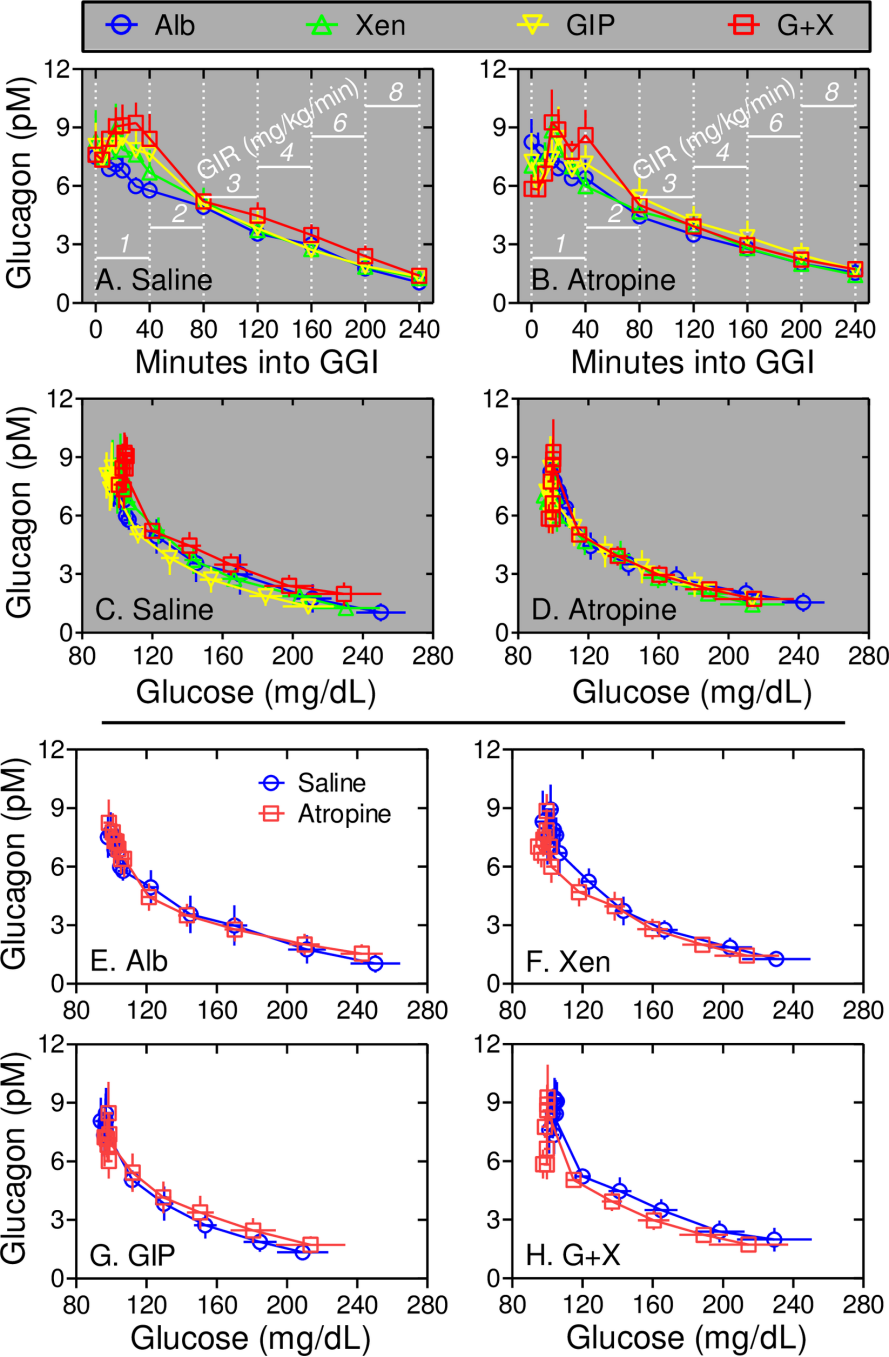
Xen amplifies the rapid and transient response to GIP via a muscarinic receptor-independent mechanism. Panels A and B: Plasma glucagon levels were measured at the indicated times during graded glucose infusions (GGIs) with the indicated peptide(s) and with the saline infusion (Panel *A*) or atropine infusion (Panel B). The glucose infusion rate (GIR) for each 40 minute step is shown in white. Note the rapid and transient increase in glucagon levels during peptide infusions only from 0 to 40 minutes. Panels C and D: Plasma glucagon levels during graded glucose infusions with saline (Panel C) or atropine (Panel D) infusions were plotted as a function of plasma glucose levels. Panels E-H: Plasma glucagon and glucose levels were determined during graded glucose infusions with Albumin alone (Panel E), Xen alone (Panel F), GIP alone (Panel G), and the combination of GIP plus Xen (Panel H). Plasma glucagon versus glucose values during infusion of saline or atropine are shown for each peptide(s). Note that atropine had little effect on the rapid and transient (0 to 40 min) or on the glucose-regulated (40–240 min) glucagon responses regardless of the peptide administered.

**Fig 9:**
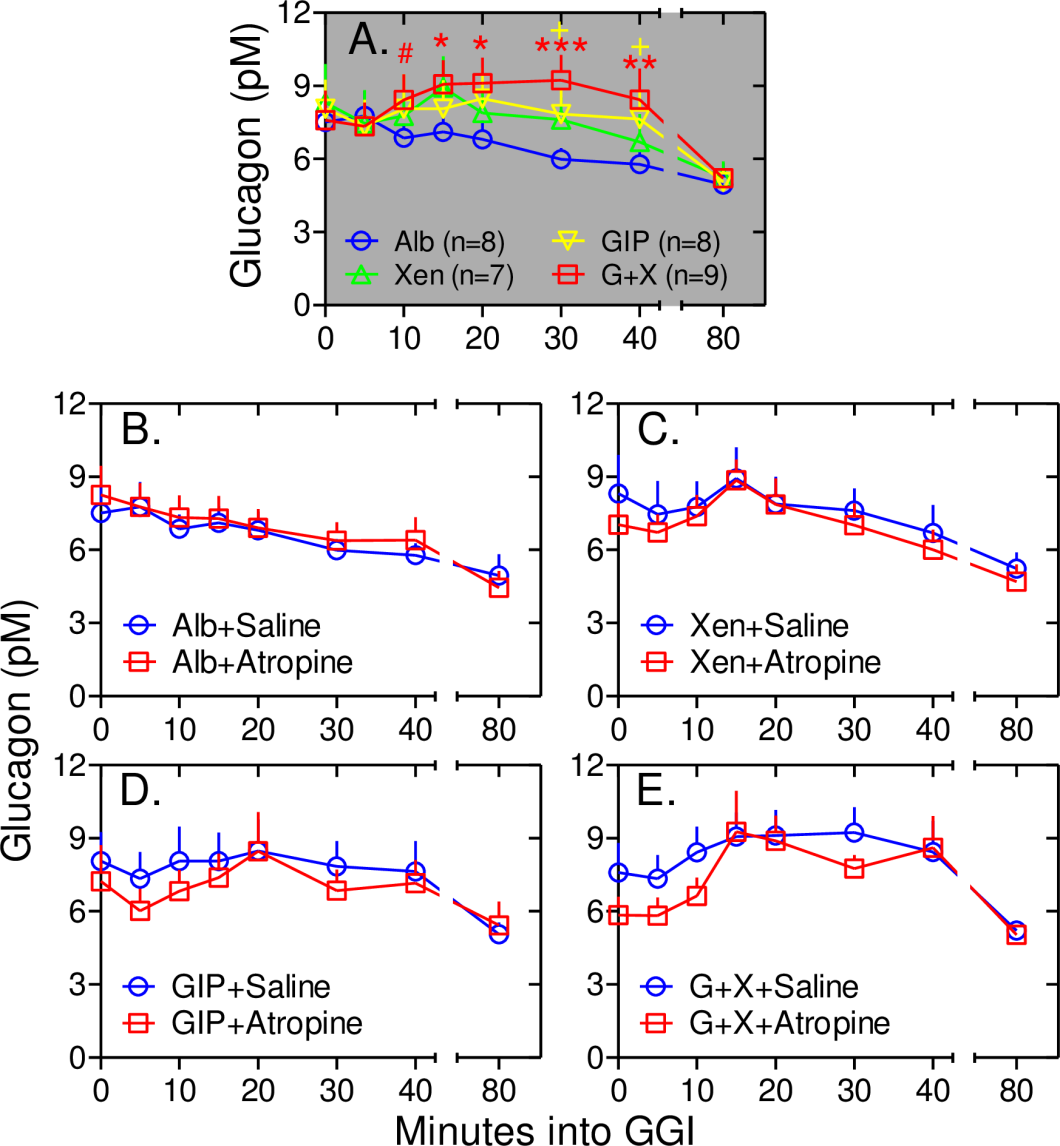
Atropine does not inhibit the glucagon response to peptides. Data from Fig 8A and 8B representing the rapid and transient responses (0 to 40 minutes) were replotted. Panel A: Note that compared to albumin alone, the increase in plasma glucagon is highly significant during infusion of GIP+Xen from 5 to 40 minutes. Red #, *, **, and *** represent *p* <0.03, <0.01, <0.001, and <0.0001, respectively compared to albumin alone. The yellow + sign indicates a *p* value <0.07 for GIP alone compared to albumin alone. Panels B-E. Data from graded glucose infusions with albumin alone (Panel *B*), Xen alone (Panel C), GIP alone (Panel D) and GIP+Xen (Panel E) are shown for infusions with (red squares) and without (blue circles) atropine. Compared to the saline control, atropine had no statistically significant effect on the glucagon response to any of the 4 peptide treatments.

Our earlier study showed that GIP transiently increases plasma glucagon levels as well as ISRs over the first 40 minutes of the graded glucose infusion and these responses were amplified by Xen only in subjects without type 2 diabetes mellitus [17]. Thus, the effects of peptides and atropine on plasma glucagon levels were determined (Figs 8 and 9). As in our earlier study, infusion of GIP alone increased plasma glucagon levels at the 40 min time point after starting the graded glucose infusion (*p* = 0.07) and the glucagon response at the 40 min time point was further enhanced when Xen was infused along with the GIP (*p*<0.001; Figs 8A and 9A). In the current study, additional samples were collected from 0 to 40 minutes to define this early glucagon response in greater detail. During infusion with albumin alone, plasma glucagon levels progressively declined starting as soon as the graded glucose infusion was initiated (Fig 9A and 9B). As noted for ISRs, infusion with GIP+Xen caused a rapid and transient increase in plasma glucagon levels (Fig 9A). This response was highly significant from 5 to 40 minutes after starting the graded glucose infusion when compared to that with albumin alone. The rapid glucagon responses to GIP alone and Xen alone were smaller and neither reached statistical significance (Fig 9A). Infusion of atropine did not affect plasma glucagon levels during infusion of albumin alone or any peptide(s) (Fig 9B–9E). From 40 to 240 minutes, plasma glucagon levels progressively decreased as plasma glucose levels increased (Fig 8). This decrease was observed regardless of which peptide(s) was administered indicating that it may be a glucose-regulated rather than a peptide-dependent response. As with the early response, the 40 to 240 minute glucagon responses to albumin and peptides were unaffected by atropine (Fig 9). These results indicate that cholinergic signaling does not mediate the effects of peptides on the glucagon response.

## Discussion

Transmitters and peptides released from neurons that innervate islets play important roles in regulating insulin and glucagon release [36,37]. In general, parasympathetic and sympathetic neurons that innervate pancreatic islets increase and inhibit insulin release, respectively [36–39]. In rodents, islets are richly innervated by parasympathetic neurons [40]. Many studies with genetically modified mice and/or islets indicate that cholinergic signaling via M3 muscarinic acetylcholine receptors plays an important role in regulating insulin and glucagon release [41–46]. Consistent with these results, we previously showed in hyperglycemic mice that Xen, with GIP but not alone, indirectly amplifies insulin secretion by initiating a cholinergic neural relay to islets [11]. As shown earlier [14,17] as well as in this paper, Xen amplifies the effects of GIP on insulin, glucagon, and pancreatic polypeptide release during graded glucose infusions in humans without type 2 diabetes mellitus. Although atropine completely inhibited the pancreatic polypeptide responses to Xen alone, GIP alone, and the combination of GIP plus Xen, it had little effect on rapid and transient (0 to 40 minutes) and glucose-regulated (40 to 240 minutes) insulin and glucagon responses, regardless of peptide(s) administered. Consistent with these results, we recently demonstrated that bethanechol, a muscarinic acetylcholine agonist that works only in the periphery, increased the postprandial pancreatic polypeptide response in humans with impaired glucose tolerance but had no effect on ISRs or plasma glucagon levels [16]. Similarly, Xen infusion increased the postprandial pancreatic polypeptide response 6-fold in humans with normal glucose tolerance, impaired glucose tolerance, and type 2 diabetes mellitus [16] without affecting ISRs or plasma glucagon levels [15]. Thus, our collective studies strongly suggest that cholinergic signaling plays an important role in regulating insulin and glucagon release in mice, but not humans.

An immunohistochemical study suggested that mouse, but not human, islets are richly innervated with parasympathetic neurons [40]. However, studies with the isolated perfused human pancreas have shown that electrical stimulation of the splanchnic nerve in the presence and absence of selective neural inhibitors increases both cholinergic and sympathetic input to islets which in turn, regulates insulin, glucagon, pancreatic polypeptide, and somatostatin release [47–52]. Further, neurotransmitters can regulate insulin release in isolated human islets [53]. Thus, functional neural/cholinergic signaling can regulate islet physiology in humans. This also suggests that under physiologic conditions and in the intact human, insulin secretion and glucagon release are regulated by numerous factors and studying the effects of single components in isolation may not reflect what happens in whole person physiology.

The specific pathway(s) that mediates the effects of Xen on islet physiology in humans is unknown. We have shown that neurotensin receptor-1, the major receptor for Xen, is present on nerves, but not endocrine cells, in the human pancreas [14]. In addition to parasympathetic neurons, Kirchgessner and Gershon have described an extensive network of enteric neurons that directly connect the proximal small intestine and pancreas [54–57]. These neurons can function independently of the central nervous system and are capable of modifying pancreatic islet function. We have shown that Xen excites a small subset of enteric neurons [10] raising the possibility that enteric neurons may play an underappreciated but important role for regulating islet physiology. It will be important to determine the specific neural or non-neural pathway that mediates the effects of Xen because this pathway is blunted in type 2 diabetes.

It is generally accepted that as plasma glucose levels rise, insulin secretion increases while glucagon release decreases. Our previous [15,17,32] and current studies indicate that the relationship between insulin and glucagon release is more complicated than this. For examples: i) insulin secretion and glucagon release are both rapidly and transiently increased while plasma glucose levels remain nearly euglycemic during the first 40 minutes of graded glucose infusions with GIP alone or GIP+Xen (Figs 6–9; [17]). Although the glucose concentration within the islets during the start of the graded glucose infusion is unknown, our results strongly suggest that this rapid response to peptides is largely independent of circulating plasma glucose levels. Consistent with this hypothesis, others have shown that GIP can increase glucagon release during euglycemia [58]. ii) plasma glucagon levels and ISRs both increase for the first 30 minutes after ingestion of a liquid mixed meal in humans with normal glucose tolerance, impaired glucose tolerance, and type 2 diabetes mellitus [15]; and iii) the paradoxical postprandial increases in both insulin and glucagon levels are greatly exaggerated in humans with prior Roux-en-Y gastric bypass [32]. These results suggest that the early release of both insulin and glucagon may be critical for maintaining postprandial glucose homeostasis distinct from their individual and well-established respective roles for increasing glucose clearance and augmenting glucose production. Intriguingly, the combination of insulin plus glucagon has been shown to synergistically increase hepatic FGF21 mRNA levels and peptide release [59]. FGF21 has numerous beneficial metabolic effects in both rodents and primates [60,61]. The FGF21 response in hepatocytes occurs over several hours rather than minutes [59] suggesting it could mediate delayed responses to the rapid increases in both insulin and glucagon. Additional signaling molecules are likely to be regulated by the combination of insulin plus glucagon and these factors could play important roles in rapid as well as longer-term metabolic responses.

Two limitations to our study should be addressed. First, our mouse studies suggested that atropine would completely block the effect of Xen on GIP-mediated ISRs and glucagon levels [11]. Thus, our human study was not powered to detect small effects of atropine on ISRs and plasma glucagon levels. However, atropine increased resting heart rate and completely blocked the ability of Xen to increase pancreatic polypeptide release. Thus, the atropine dosing was effective and muscarinic receptor signaling is most likely involved in only a subset of responses to Xen. Second, the current study was performed using graded glucose infusions in humans with impaired glucose tolerance. Thus, it is possible that other techniques or patient populations would yield different results. However, as previously discussed, Xen infusions and/or oral bethanechol also increased postprandial cholinergic signaling in humans with normal glucose tolerance, impaired glucose tolerance, and type 2 diabetes mellitus without affecting ISRs or glucagon levels [16]. Thus, our results appear to be generally relevant but may not be valid for all pathological conditions.

## Conclusions

In spite of the study limitations, our results indicate that in contrast to mice, cholinergic signaling plays a minor role in regulating ISRs and glucagon levels in humans and further illustrate the importance of studying islet physiology in vivo and in humans. It will be important to identify the neurotransmitters or neuropeptides that mediate the effects of Xen on insulin and glucagon release in humans because signaling pathways initiated by Xen rather than GIP fail early during the development of type 2 diabetes mellitus.

## Supporting information

S1 Appendix Original IRB approval for study. This is the original protocol as approved by our Institutional Review Board.
(DOCX)

S2 Appendix Individual data points for the atropine study. Subject characteristics were obtained during a screening visit. Heart rate as well as plasma levels of glucose, C-peptide, glucagon, pancreatic polypeptide, GIP, and xenin-25 were measured during the graded glucose infusions. Insulin secretion rates were calculated from C-peptide levels. Individual data points that were used for calculating means, AUCs, iAUCs and respective standard deviations, and/or standard error of the means are listed.
(XLSX)

S3 Appendix Trend Checklist. Page numbers in the completed Trend Checklist correspond to those in the original manuscript.
(PDF)
