# Supplementary material for: Cholinergic signaling mediates the effects of xenin-25 on secretion of pancreatic polypeptide but not insulin or glucagon in humans with impaired glucose tolerance
Source: PLoS One. 2018 Feb 21;13(2):e0192441. doi: 10.1371/journal.pone.0192441 (PMC5821323; doi:10.1371/journal.pone.0192441)
Supplement: S1 Appendix — This is the original protocol as approved by our Institutional Review Board. (DOCX) [file pone.0192441.s001.docx]

**S1 Appendix. Original IRB approved protocol for study.**

***Protocol 10 is designed to determine if a cholinergic relay mediates the effects of Xen on insulin secretion rates (ISRs) and plasma glucagon levels in humans.*** We previously showed that xenin-25 (Xen): i) in mice, increases the effects of GIP on insulin release via a cholinergic relay independent from the CNS that requires mAChRs in the periphery [1] and ii) in humans, increases the effect of GIP on ISR and plasma glucagon levels and these effects are greatest in humans with IGT, lower in those with NGT, and lost in T2DM [2]. Infusion with GIP plus xenin-25 (G+X) also increases cholinergic input to islets equally well in humans with NGT, IGT, or T2DM. *Thus, it is important to determine if the Xen-induced cholinergic input to islets is responsible for the increase in GIP-mediated ISR and plasma glucagon levels. If so, the* ***response*** *to cholinergic input is a major defect in humans with T2DM and would provide important and novel insights into the development of T2DM in humans.* Atropine inhibits mAChRs and will inhibit the effects of Xen on GIP-mediated ISR and plasma glucagon levels if, as in mice, mAChRs relay the Xen effects.

**Experimental Design** is essentially the same as described for the graded glucose infusions (GGIs) in Study Protocol 1 [2] except a) only subjects with IGT will be studied; b) subjects will be infused with only 4 of the peptides/albumin (see below); and c) each peptide/albumin study will be conducted with and without infusion of atropine sulfate (thus, 8 total GGIs). To monitor for symptoms of atropine toxicity, subjects will be placed on continuous cardiac monitoring with blood pressure and heart rate checked every 15 minutes. Monitoring will continue for at least 2 hour after the atropine infusion is discontinued. Participants will be released at this time as long as their anti-cholinergic symptoms have decreased. The study will be stopped in the event of tachycardia (>130 beats per minute) or paradoxical bradycardia (<60 beats per minute) or other cardiovascular reactions (e.g. arrhythmia, hyper- or hypotension), changes in mental status (e.g. confusion or dizziness), or dyspnea. Any participant experiencing severe adverse reaction to atropine will also be dropped from the study.

**Screening:** Subjects will be recruited through Washington University’s Research Participant Registry, our own database, and by visibility on the Clinicaltrials.gov website. Based on our previous 3 studies, most subjects are not aware of having IGT and we will have to screen ~10 subjects to identify 1 with IGT (2-h glucose = 140-199 mg/dL). Thus, during an initial visit, fasted subjects without T2DM will i) be consented, ii) complete a health history questionnaire, and iii) have blood drawn for screening labs including plasma glucose, hematocrit, hemoglobin, HbA1C, Lipid Panel, electrolytes, amylase, and thyroid, liver and kidney function. Those with impaired fasting glucose (110 to ≤126 mg/dL) and otherwise eligible will receive the screening OGTT on a subsequent visit to determine final eligibility. ***The use of atropine is contraindicated in people with narrow-angle glaucoma, obstructive uropathy including benign prostatic hypertrophy, pyloric stenosis, myasthenia gravis, asthma, hyperthyroidism, angina and cardiac arrhythmias including heart block. In addition to general exclusion criteria, subjects with any of these conditions will also be excluded from this study.***

**Power calculations** are based on our previous results [2] where infusion of G+X produces a 50% increase in the effects of GIP on ISR in subjects with IGT. The primary hypothesis is that this effect of Xen will be blocked by atropine. Thirteen subjects will provide 81% power to detect a 78% decrease in this Xen effect. To allow for a 20% drop out rate, 16 subjects will be enrolled. Similar power applies to glucagon measurements. Based on preliminary results and results from others [3], atropine should completely block the Xen-induced PP response. Ten subjects will provide 89% power to detect a 55% decrease in the PP response. Thus, loss of the PP response would be easily detectable in our study.

**Dosing** for glucose, albumin alone (no peptide), GIP alone, xenin-25 alone, and G+X are as previously approved by HRPO and the FDA for Protocol 1 and used in Study Protocol 1. Only the lower dose of peptides (4 pmoles/kg/min) will be administered. Dosing for atropine sulfate is based on a survey of the literature [4–15] and Clinicaltrials.gov website (NCT00468091; NCT00689208; and NCT00992901) for studies similar to ours. The FDA has previously approved the use of atropine with our meal tolerance test in conjunction with infusion of xenin-25 (See Appendix 6 of our FDA Annual Report; note that this is also a slightly higher dose than we are now proposing to administer). Atropine sulfate will be administrated by primed-continuous IV infusion starting at minus 30 minutes (priming dose of 0.4 mg/m^2^ over 2 minutes followed by continuous dose of 0.3 mg/m^2^/hour). Based on the literature, this dose is expected to increase resting heart rate ~25 beats per minutes and should be well-tolerated. Saline alone will be infused when atropine is not administered.

**Graded Glucose Infusions (GGIs):** All subjects will receive 8 separate GGIs as outlined below, each on a separate occasion. Subjects will be blinded to treatment. Visits 1, 3, 5, and 7 will be randomized with respect to peptide. Visits 2, 4, 6, and 8 will be conducted with the same peptide infused during visits 1, 3, 5, and 7, respectively except saline instead of atropine (or vice versa) will be infused. This will ensure that in case of drop outs, matched infusions (each peptide ± atropine) are obtained for each subject. Sampling will begin 15 minutes after the IVs are in place (which equals the minus 50 min time point). Based on our previous results with Protocol 1, 5 mL of blood will be drawn for measurements of plasma glucose and preparation of heparinized plasma at the following time points (in minutes): -50, -40, -30, -20, -10, 0, 5, 10, 15, 20, 30, 40, 50, 60, 70, 80, 90, 100, 110, 120, 130, 140, 150, 160, 170, 180, 190, 200, 210, 220, 230, 240. An additional 4 mL of blood will collected for preparation of EDTA/Trasylol plasma at -50, -40, -30, -20, -10, 0, 5, 10, 15, 20, 30, 40, 80, 120, 160, 200, and 240. Total blood collected during the GGI is ~228 mL. With 5 mL for post GGI (recovery) glucose measurements, a maximum of 700 mL blood will be collected from any individual within 9 consecutive weeks (3 visits/9 weeks). Based on Hb levels measured in our previous studies, this should not present a risk to the participants. In addition, Hb levels are measured immediately before each study visit and anyone with a Hb <11.2%mg/dl will have that study delayed.

Visit 1. A GGI will be conducted with infusion of **saline** and Alb.

Visit 2. A GGI will be conducted with infusion of **atropine** and Alb.

Visit 3: A GGI will be conducted with infusion of **saline** and Xen.

Visit 4: A GGI will be conducted with infusion of **atropine** and Xen.

Visit 5: A GGI will be conducted with infusion of **saline** and GIP.

Visit 6: A GGI will be conducted with infusion of **atropine** and GIP.

Visit 7: A GGI will be conducted with infusion of **saline** and G+X.

Visit 8: A GGI will be conducted with infusion of **atropine** and G+X.

**Approach to Data Analysis and Anticipated Results.**

Data analysis is essentially as described in our publication of results from Study Protocol 1 [2].

Reference List

1. Wice BM, Wang S, Crimmins DL, Diggs-Andrews KA, Althage MC, Ford EL et al. (2010) Xenin-25 potentiates glucose-dependent insulinotropic polypeptide action via a novel cholinergic relay mechanism. J Biol Chem 285: 19842-19853.

2. Wice BM, Reeds DR, Tran H, Crimmins DL, Patterson BW, Dunai J et al. (2012) Xenin-25 Amplifies GIP-Mediated Insulin Secretion in Humans with Normal and Impaired Glucose Tolerance, but not Type 2 Diabetes. Diabetes 61: 1793-1800.

3. Fletcher DR, Shulkes A, Bladin PH, Hardy KJ (1983) The effect of atropine on bombesin and gastrin releasing peptide stimulated gastrin, pancreatic polypeptide and neurotensin release in man. Regul Pept 7: 31-40.

4. Schneeberger D, Tappy L, Temler E, Jequier E (1991) Effects of muscarinic blockade on insulin secretion and on glucose-induced thermogenesis in lean and obese human subjects. Eur J Clin Invest 21: 608-615.

5. Teff KL, Townsend RR (1999) Early phase insulin infusion and muscarinic blockade in obese and lean subjects. Am J Physiol 277: R198-R208.

6. Teff KL, Alavi A, Chen J, Pourdehnad M, Townsend RR (1999) Muscarinic blockade inhibits gastric emptying of mixed-nutrient meal: effects of weight and gender. Am J Physiol 276: R707-R714.

7. Teff KL, Townsend RR (2004) Prolonged mild hyperglycemia induces vagally mediated compensatory increase in C-Peptide secretion in humans. J Clin Endocrinol Metab 89: 5606-5613.

8. Vozarova de Court, Weyer C, Stefan N, Horton M, DelParigi A, Havel P et al. (2004) Parasympathetic blockade attenuates augmented pancreatic polypeptide but not insulin secretion in Pima Indians. Diabetes 53: 663-671.

9. Boyle PJ, Liggett SB, Shah SD, Cryer PE (1988) Direct muscarinic cholinergic inhibition of hepatic glucose production in humans. J Clin Invest 82: 445-449.

10. Parkman HP, Trate DM, Knight LC, Brown KL, Maurer AH, Fisher RS (1999) Cholinergic effects on human gastric motility. Gut 45: 346-354.

11. Adner N (1990) Influence of naloxone, atropine, and metoclopramide on ethanol augmentation of insulin secretion after intravenous glucose stimulation. Pancreas 5: 460-466.

12. Lidums I, Hebbard GS, Holloway RH (2000) Effect of atropine on proximal gastric motor and sensory function in normal subjects. Gut 47: 30-36.

13. Schirra J, Nicolaus M, Woerle HJ, Struckmeier C, Katschinski M, Goke B (2009) GLP-1 regulates gastroduodenal motility involving cholinergic pathways. Neurogastroenterol Motil 21: 609-e22.

14. Balks HJ, Holst JJ, von zur MA, Brabant G (1997) Rapid oscillations in plasma glucagon-like peptide-1 (GLP-1) in humans: cholinergic control of GLP-1 secretion via muscarinic receptors. J Clin Endocrinol Metab 82: 786-790.

15. Schvarcz E, Palmer M, Aman J, Berne C (1995) Atropine inhibits the increase in gastric emptying during hypoglycemia in humans. Diabetes Care 18: 1463-1467.
